# Job-Sharing in General Practice—A Thing of Beauty

**Published:** 1991-09

**Authors:** William Hamilton

**Affiliations:** General Practitioner, Exeter


### Job-Sharing in General Practice — a thing of beauty?

**William Hamilton, MRCP, MRCGP**
General Practitioner, Exeter.

The imposition of the new General Practice contract, with all the acrimony it has engendered, has obscured another change in General Practice, which has far-reaching potential. This is the explosive rise in job-sharing seen recently, exemplified by the figures for Devon FHSA, which has seen a rise from 4 job-sharing GPs, in two years, to 28 by June, 1991 (personal communication, Devon FHSA).

In this article, I propose to review the mechanics of job-sharing, the financial implications, the reasons for (and against!), and to attempt a prediction of the future.

### WHAT IS A JOB-SHARE?

This is when two GPs undertake to manage a single list, dividing the duties as they wish. It is quite distinct from two part-time GPs, both in the organisation of the practice, and the financial implications. The FHSA regard the pair, to all intents and purposes as one unit, and responsibility within the unit is for the two doctors to apportion, with the proviso that the FHSA is informed as to the hours each GP is working.

There are three broad categories at present:

1) The experienced GP who plans retirement soon (or has taken 24 hour retirement), and wishes to reduce the number of hours he works, particularly at night. The new job-sharer undertakes all the night work, an agreed amount of surgery work, and the finances are divided appropriately. Usually, the new partner expects to take over the whole list on his job-sharer's retirement (or second retirement) and in the meantime will do other medical work outside the practice to boost his earnings. This is a classic symbiosis, with the rejuvenated senior partner passing on the tips of the trade, while the junior partner gets his foot in the door a few years early.

2) The husband and wife team. The chief attraction is for those with young families, where the parents can share the domestic as well as the medical work. Liaison between the two GPs should be easy, as should be division of the remuneration, but they must exercise good self discipline to allow themselves time at home without medicine rearing its ugly head! The other members of the practice are often chary of appointing such a job-share, for fear the couple will dominate, always voting in the same way. This can usually be circumvented by establishing a voting structure which allows job-sharers only half a vote each. Furthermore, what husband and wife have ever done this?

3) Both job-sharers have long-term commitments outside the practice. These are frequently domestic, but those with academic or political posts will fall into this category.

The majority of job-sharers in all three categories arise by a full-time GP reducing his hours, but increasingly job-sharers have been appointed to vacancies advertised as full-time. I feel this trend will increase, particularly with the increase in female graduates in the last decade. For a doctor with domestic commitments, a job-share is a method of working part-time with far more job security and flexibility than an assistant post or part-time partnership.

The BMA keeps a register of potential GP job-sharers, although most pairs are arranged locally, by two GPs who know each other beforehand.

### FINANCES

Only basic guidelines can be given, as division of the duties, and the remuneration is for the job-sharers, and their partners to decide. Frequently, only one job-sharer does the on-call duties and so the practice profits share will have to reflect this. The distribution of income generated privately, or from posts outside the practice will have to be settled, particularly as job-sharers have more time than full-time partners to earn extra.

As far as the FHSA are concerned, all allowances are given as if the pair were one GP — except occasionally when both offer 19-26 hours availability, when three-quarter basic practice allowance may be claimable by each. The only allowance definitely obtainable in full by each job-sharer is PGEA; all others are halved. The pair are counted together for assessment of target payments. A check must be made that both GPs only count as one for the purposes of earning a higher rate night visit fee — it is possible that if both counted, then more than 10 GPs would be in the rota, jeopardising everyone's fees.

### PROS AND CONS

The flexibility of job-sharing provides many advantages for the individual GPs, but also some disadvantages. The major gains are

More time away from the practice during the working week
Flexibility of working hours from week to week
Time during working hours for administrative work, or outside interests
Reduction in out of hours work
Less stress — because time pressures are less

However, this must be counterbalanced by

Less earnings from the practice
More need for organisation of responsibilities i.e. Who sees the results, letters etc? Who accepts or rejects patients?
The need to liaise between job-sharers
Potential differences of opinion in clinical care
Parkinson's Law — I have yet to meet a part-time GP who does not feel that they do more than their strict share — and job-sharers are no exception.

For the practice, there are also bonuses and problems with having a job-share instead of a single partner, such as there is an inbuilt locum facitity; with advance notice one of the job-sharers may be able to work full-time, or cover for absent colleagues
in a crisis more hands are on board to help
regular administrative tasks can be spread over more people, reducing the individual burden but, there will be more paperwork and more people at partnership meetings
the other members of the primary health care team will need to know who is individually responsible for each patient (usually this is self-evident, only one of the pair being present, but a useful rule of thumb is to regard the last person to see the patient as being in charge).

Where does this leave the poor patient? Surprisingly few seem confused by the new status quo, especially if the two job-sharers spread their availability over most of the week, so that the patient can see one of the pair easily. Most are able rapidly to use the extra flexibility, almost like having an inbuilt second opinion, although this could be detrimental if this led to dilution, or alteration of medical advice. Furthermore, they will probably have to see less locums, their GP will be less tired, and may even be able to offer them more time.

### THE FUTURE

There has certainly been an increase in job-sharing in Devon — amazingly the Department of Health do not know the figures for the country as a whole, but the pattern is likely to be countrywide. There are reasons for this increase; those enumerated above, plus the increase in female graduates in the 1980s, and the increased work with the new contract, which has swung the balance to job-sharing in some older GPs.

A modern GPs life is like trying to squeeze a quart of life into a pint pot — with a job-share the quart becomes smaller, and the pot bigger.

Correspondence to: 12 Barnfield Hill, Exeter EX1 1SR.

